# An MRI-based strategy for differentiation of frontotemporal dementia and Alzheimer’s disease

**DOI:** 10.1186/s13195-020-00757-5

**Published:** 2021-01-12

**Authors:** Qun Yu, Yingren Mai, Yuting Ruan, Yishan Luo, Lei Zhao, Wenli Fang, Zhiyu Cao, Yi Li, Wang Liao, Songhua Xiao, Vincent C. T. Mok, Lin Shi, Jun Liu, the National Alzheimer’s Coordinating Center, the Alzheimer’s Disease Neuroimaging Initiative, the Frontotemporal Lobar Degeneration Neuroimaging Initiative, Howard Rosen, Bradford C. Dickerson, Kimoko Domoto-Reilly, David Knopman, Bradley F. Boeve, Adam L. Boxer, John Kornak, Bruce L. Miller, William W. Seeley, Maria-Luisa Gorno-Tempini, Scott McGinnis, Maria Luisa Mandelli

**Affiliations:** 1 Department of Neurology, Sun Yat-sen Memorial Hospital, Sun Yat-sen University, No. 107 Yanjiang West Road, Guangzhou, Guangdong China; 2 BrainNow Research Institute, Shenzhen, China; 3 Gerald Choa Neuroscience Centre, Lui Che Woo Institute of Innovative Medicine, Division of Neurology, Department of Medicine and Therapeutics, The Chinese University of Hong Kong, Hong Kong SAR, China; 4 Department of Imaging and Interventional Radiology, The Chinese University of Hong Kong, Hong Kong SAR, China; 5 Guangdong Province Key Laboratory of Brain Function and Disease, Zhongshan School of Medicine, Sun Yat-sen University, Guangzhou, China; 6 Laboratory of RNA and Major Diseases of Brain and Heart, Sun Yat-sen Memorial Hospital, Sun Yat-sen University, Guangzhou, China

**Keywords:** Frontotemporal dementia, Alzheimer’s disease, Structural magnetic resonance imaging, AD resemblance atrophy index, Frontotemporal dementia index

## Abstract

**Background:**

The differential diagnosis of frontotemporal dementia (FTD) and Alzheimer’s disease (AD) is difficult due to the overlaps of clinical symptoms. Structural magnetic resonance imaging (sMRI) presents distinct brain atrophy and potentially helps in their differentiation. In this study, we aim at deriving a novel integrated index by leveraging the volumetric measures in brain regions with significant difference between AD and FTD and developing an MRI-based strategy for the differentiation of FTD and AD.

**Methods:**

In this study, the data were acquired from three different databases, including 47 subjects with FTD, 47 subjects with AD, and 47 normal controls in the NACC database; 50 subjects with AD in the ADNI database; and 50 subjects with FTD in the FTLDNI database. The MR images of all subjects were automatically segmented, and the brain atrophy, including the AD resemblance atrophy index (AD-RAI), was quantified using AccuBrain®. A novel MRI index, named the frontotemporal dementia index (FTDI), was derived as the ratio between the weighted sum of the volumetric indexes in “FTD dominant” structures over that obtained from “AD dominant” structures. The weights and the identification of “FTD/AD dominant” structures were acquired from the statistical analysis of NACC data. The differentiation performance of FTDI was validated using independent data from ADNI and FTLDNI databases.

**Results:**

AD-RAI is a proven imaging biomarker to identify AD and FTD from NC with significantly higher values (*p* < 0.001 and AUC = 0.88) as we reported before, while no significant difference was found between AD and FTD (*p* = 0.647). FTDI showed excellent accuracy in identifying FTD from AD (AUC = 0.90; SEN = 89%, SPE = 75% with threshold value = 1.08). The validation using independent data from ADNI and FTLDNI datasets also confirmed the efficacy of FTDI (AUC = 0.93; SEN = 96%, SPE = 70% with threshold value = 1.08).

**Conclusions:**

Brain atrophy in AD, FTD, and normal elderly shows distinct patterns. In addition to AD-RAI that is designed to detect abnormal brain atrophy in dementia, a novel index specific to FTD is proposed and validated. By combining AD-RAI and FTDI, an MRI-based decision strategy was further proposed as a promising solution for the differential diagnosis of AD and FTD in clinical practice.

**Supplementary Information:**

The online version contains supplementary material available at 10.1186/s13195-020-00757-5.

## Introduction

Frontotemporal dementia (FTD) is one of the main causes of dementia in people under 65, which accounts for nearly 20% of neurodegenerative dementia [1]. Clinical symptoms of FTD overlap with other types of dementia, psychiatric disorders, or Parkinson’s disease. Alzheimer’s disease (AD) is the most common type of dementia and is often difficult to differentiate with FTD, especially in the early stage [2, 3]. Currently, there are no disease-modifying treatments for FTD. Acetylcholinesterase inhibitors widely used in patients with AD could lead to worsening of symptoms in those with FTD [4]. Therefore, accurate diagnosis of FTD and AD may avoid severe consequence in treatment outcome. In addition to clinical practice, the reduction of misdiagnosis is of utility in the differentiation for clinical trials, as well [5].

Structural magnetic resonance imaging (sMRI) is a common non-invasive examination in the diagnosis of dementia. Compared with computed tomography, positron emission tomography, and advanced MRI sequences, sMRI is advantageous in being widely accessible, free of ionizing or nuclear radiation, objective for visualizing brain atrophy, and with good tissue contrast [6]. In clinical practice, neurologists and radiologists use various visual rating scales to evaluate the degree of brain atrophy in MR images [7, 8]. In spite of being convenient to perform, visual rating is highly subjective and dependent on experience which is prone to lead to misdiagnosis and missed diagnosis in practice [9]. Recently, automatic brain segmentation, quantitative analysis, and machine learning have been studied a lot in neurodegenerative diseases [10–18]. However, plenty of machine learning-based classification highly rely on limited training data and lack of underlying clinical rationale [19–22]. Because it is hard for clinicians to understand how and why these artificial intelligence methods made a certain decision, the lack of interpretability limits their applicability in clinical practice [23]. Secondly, most studies were based on samples from a single center and may not be translated to other centers because of the heterogeneity in diagnostic criteria, imaging parameters, and sociodemographic characteristics of patients. Finally, many studies only focus on distinguishing AD from behavioral variant frontotemporal dementia (bvFTD) [24, 25]. But, in fact, due to the presence of either mild or overlapping linguistic deficits, AD with speech impairment can also be confused with the subtypes of FTD, such as primary progressive aphasia (PPA) including semantic variant PPA (svPPA) and non-fluent variant PPA (nfvPPA) [26]. Therefore, it is theoretically desirable and practically meaningful if AD can be differentiated with FTD spectrum disorders.

The primary objective of this study is to investigate the MRI volumetric and atrophic patterns among FTD, AD, and normal controls (NC) collected from multicenter data. Based on these findings, the secondary objective is to derive an atrophy index that is specific to reflect FTD brain atrophy, named FTD index (FTDI). FTDI was an individualized index derived from brain regions with significant volume difference between FTD and AD, which were identified by group-level comparisons, and the differentiation performance of the proposed index was validated using multicenter data from another two databases.

## Materials and methods

### Subjects

In this retrospective study, all the subjects were included from the National Alzheimer’s Coordinating Center (NACC), the Alzheimer’s Disease Neuroimaging Initiative (ADNI), and the Frontotemporal Lobar Degeneration Neuroimaging Initiative (FTLDNI) databases (see Additional Material 1). The data are the result of collaborative efforts at three sites in North America.

**Fig. 1:**
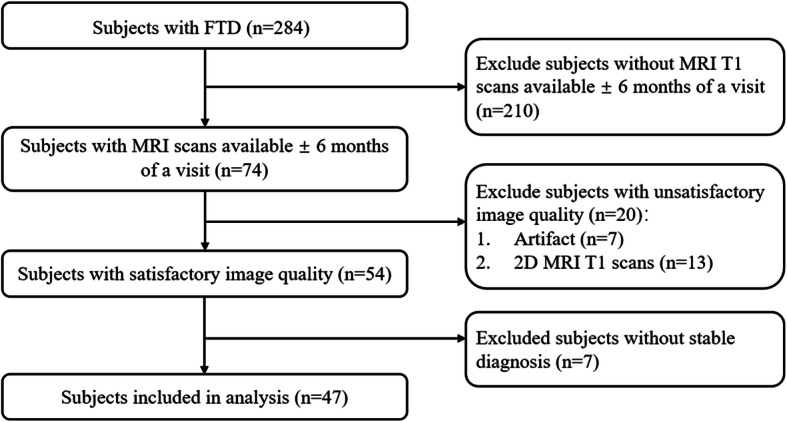
Subject screening from the NACC database

We first included 47 subjects with FTD (19 bvFTD, 12 svPPA, 2 nfvPPA, and 14 not otherwise specified) from NACC. Then, 47 subjects with probable AD and 47 NC perfectly matching those with FTD (Table 1) in age, educational level, gender, race, and global CDR score (except for NC) using the CDR® Dementia Staging Instrument plus NACC FTLD Behavior & Language Domains [27–29] were selected from NACC as well. Subjects were included if their diagnoses were stable (> 1 year) and 3D T1-weighted MRI scans were available ± 6 months of a visit. Scans containing large artifacts were excluded by visual inspection (Fig. 1). After that, as a validation dataset, 50 subjects with AD in the ADNI database and 50 subjects with FTD (20 bvFTD, 10 svPPA, 10 nfvPPA, and 10 not otherwise specified) in the FTLDNI database were included in this study.

**Table 1:** Demographic characteristics of subjects included

**NACC**	**FTD (*n*** **= 47)**	**AD (*n*** **= 47)**	**NC (*n*** **= 47)**	***p*** **value**
Age (years), mean (SD)	64.55 (10.15)	64.55 (10.28)	64.57 (10.13)	> 0.999
Education (years), mean (SD)	15.45 (2.98)	15.62 (2.78)	15.68 (2.86)	0.920
Gender, male (*n* (%))	25 (53.2)	25 (53.2)	25 (53.2)	> 0.999
Race, White (*n* (%))	47 (100.0)	47 (100.0)	47 (100.0)	> 0.999
ICV (mL), mean (SD)	1472.14 (155.82)	1526.37 (170.79)	1505.12 (145.51)	0.247
Global CDR^				> 0.999
0.0 = no impairment (*n* (%))	6 (12.8)	6 (12.8)	47 (100.0)	–
0.5 = questionable impairment (*n* (%))	21 (44.7)	21 (44.7)	–	–
1.0 = mild impairment (*n* (%))	17 (36.2)	17 (36.2)	–	–
2.0 = moderate impairment (*n* (%))	3 (6.4)	3 (8.5)	–	–
**ADNI + FTLDNI**	**FTD (*n*** **= 50)**	**AD (*n*** **= 50)**	–	***p*** **value**
Age (years), mean (SD)	64.64 (7.09)	64.22 (7.58)	–	0.484
Education (years), mean (SD)	15.98 (3.57)	16.02 (2.56)	–	0.241
Gender, male (*n* (%))	27 (54.0)	23 (46.0)	–	0.548
Race, White (*n* (%))	45 (90.0)	46 (92.0)	–	0.727
ICV (mL), mean (SD)	1521.82 (131.45)	1456.48 (158.80)		0.070
Global CDR^			–	**0.002**
0.0 = no impairment (*n* (%))	3 (6.0)	0 (0.0)	–	–
0.5 = questionable impairment (*n* (%))	24 (48.0)	20 (40.0)	–	–
1.0 = mild impairment (*n* (%))	16 (32.0)	30 (60.0)	–	–
2.0 = moderate impairment (*n* (%))	7 (14.0)	0 (0.0)	–	–

^Data was compared between the FTD and AD groups only. *p* value would be highlighted in bold when it was below 0.05

The National Institute of Neurological and Communicative Disorders and Stroke and the AD and Related Disorders Association (NINCDS/ADRDA) criteria [30] and the National Institute on Aging-Alzheimer’s Association (NIA-AA) criteria [31] were applied for the diagnosis of probable AD. The Mckhann et al. and Rascovsky et al. criteria [32, 33] were used for bvFTD, and the Mckhann et al. and Gorno-Tempini et al. criteria [32, 34] for PPA. The inclusion criteria for the NC were normal in general physical status and CDR of 0, suggesting no cognitive impairment.

### MRI acquisition and volumetric analysis

MR images from NACC were collected following varying acquisition protocols by different centers, which reflects the real-world scenario. In the ADNI database, the subjects were scanned using 3-T scanners including GE, Philips, or Siemens scanners. For T1-weighted MRI, the inversion recovery-fast spoiled gradient recalled (IR-FSPGR) sequences were used by GE scanners, and magnetization-prepared rapid gradient echo (MP-RAGE) sequences were used by Philips and Siemens scanners. More details concerning the sMRI images are available on the ADNI homepage (http://adni.loni.usc.edu/methods/mri-tool/mri-analysis/). In the FTLDNI database, the subjects were scanned using Siemens TrioTim 3-T scanners at three centers. MP-RAGE sequence was used to acquire T1-weighted MR images [35]. All MRI scans were analyzed using AccuBrain® (BrainNow Medical Technology Limited), a fully automatic neuroanatomical volumetry tool that quantifies brain volumes of various subcortical structures, ventricles, and cortical lobar atrophy within clinically acceptable time. AccuBrain® performs brain structure segmentation based on a multi-atlas image registration scheme. The accuracy of hippocampus segmentation of AccuBrain® was validated using data from the ADNI database [36], and more technical description of AccuBrain® and its comparison with other automatic brain image segmentation schemes were described in our previous work [37–39]. Since the main objective of this study is to identify FTD from NC and AD, we focus on the brain regions known to be associated with cognition and behavior, including the brain parenchyma, typical subcortical structures (bilateral hippocampus, amygdala and caudate, etc.), ventricular regions (ventricular system, lateral ventricle, and inferior lateral ventricle), and atrophy of lobar regions (frontal, occipital, temporal, parietal, cingulate, and insular lobe). To correct for the head size difference, we took the *relative volume* and *atrophy* as our volumetric brain measures. The *relative volume* of each region was defined as the absolute volume divided by the total intracranial volume (ICV). The *atrophy* of a particular lobe was defined as the ratio between the volume of cerebrospinal fluid and brain parenchyma within that lobe. The AD resemblance atrophy index (AD-RAI), representing the similarity of the brain atrophy pattern with AD, was also calculated for each subject in this study. Calculating AD-RAI is a unique function of AccuBrain® and was previously proven to be able to identify AD and estimate the risk of cognitive decline [40, 41].

In addition to AD-RAI, in this study, a novel MRI-based biomarker named FTDI was derived based on the volumetric results of brain regions with significant volume difference between AD and FTD using the NACC data.

To increase the comparability of the numerical metrics, the relative volumes of the involved brain regions and lobe atrophy *R*_*i*_(*j*) were normalized to *R*′_*i*_(*j*), i.e.,

$$ {R}_i^{\prime }(j)=\frac{R_i(j)-\min \left({R}_i\right)}{\max \left({R}_i\right)-\min \left({R}_i\right)} $$

where *R*_*i*_(*j*) is the relative volume of the *i*th volumetric brain measure of the *j*th subject among all the subjects from NACC included in this study. For the ease of formulation, assume values in the array *R*_*i*_ are arranged so that the first *m* values are the subjects with FTD and the total number of subjects with FTD and AD is *n*. The “FTD dominant” structures are the structures with statistically larger *R*_*i*_ values in the FTD group, and the “AD dominant” structures are those with statistically larger *R*_*i*_ values in the AD group. It should be noted that the “FTD/AD dominant” structures did not refer to the abnormal brain regions of FTD/AD patients. They were derived from the group-level comparisons and were the volumetric metrics with the statistically larger values. min(*R*_*i*_) and max(*R*_*i*_) represent the minimal and maximal values in the *i*th volumetric brain measure of all subjects from NACC included in this study, respectively.

As each lobe atrophy contributed uniquely in the differentiation of AD and FTD, a weight *w*_*i*_ was calculated for each “FTD dominant” and “AD dominant” structures as the normalized absolute difference of *R*_*i*_ between FTD and AD patients:

$$ {w}_{\boldsymbol{i}}=\frac{\left|\sum \limits_{j=1}^m{R}_i(j)-\sum \limits_{j=m+1}^n{R}_i(j)\right|}{\sum \limits_{j=1}^m{R}_i(j)+\sum \limits_{j=m+1}^n{R}_i(j)} $$

In order to increase the differentiation ability, the FTDI of an individual brain is defined as the ratio between its weighted sum of *R*_*i*_ in the “FTD dominant” structures over that of the “AD dominant” structures.

$$ \mathrm{FTDI}(j)=\frac{\sum \limits_{i=1}^p{w}_i{R}_i^{\prime }(j)}{\sum \limits_{i=p+1}^q{w}_i{R}_i^{\prime }(j)} $$

where *i* ={1, …, *p*} are the “FTD dominant” structures and *i* ={*p*+1, …, *q*} are the “AD dominant” structures.

When FTDI was validated using the ADNI and FTLDNI data, the maximum and minimum values from NACC were used in the normalization and any value of *R*_*i*_ ′ (*j*) below zero would be set to zero.

### Visual assessment

All the MRI scans were viewed in the coronal plane and the severity of medial temporal lobe atrophy (MTA) was assessed visually by two experienced neurologists (YRM and YTR) according to a 5-point scale [42]. The single MTA score of each subject was the average score of left and right hemispheres with substantial agreement and high reliability between the two raters (*κ* =0.75, ICC > 0.9).

### Statistical analysis

We compared the demographic characteristics of the three groups (NC, AD, FTD) using ANOVA analysis and chi-square test. Data of volumetric brain measures were not normally distributed and therefore were analyzed statistically with the Friedman *M* test, and a *p* value below 0.05 was considered to indicate a significant difference. If significant differences were found, a post hoc analysis would be performed with the Wilcoxon signed-rank test with Bonferroni correction, and a corrected *p* value below 0.05 was considered to indicate a significant difference. Receiver operating characteristic (ROC) curves were drawn, and the area under the curve (AUC), sensitivity (SEN), and specificity (SPE) were used to verify the diagnostic efficiency of AD-RAI and FTDI. Nonnormally distributed values were described as medians with inter-quartile range. All the statistical analyses were performed with software (SPSS version 25.0, IBM, Armonk, NY).

## Results

### Demographic and clinical parameters

The characteristics (age, educational level, gender, race, and global CDR score) of subjects from NACC were matched in pairs and the changes among each group showed no significant difference (Table 1). The characteristics of subjects from ADNI and FTLDNI were also displayed (Table 1).

### AD-RAI can identify AD and FTD from NC

**Fig. 2:**
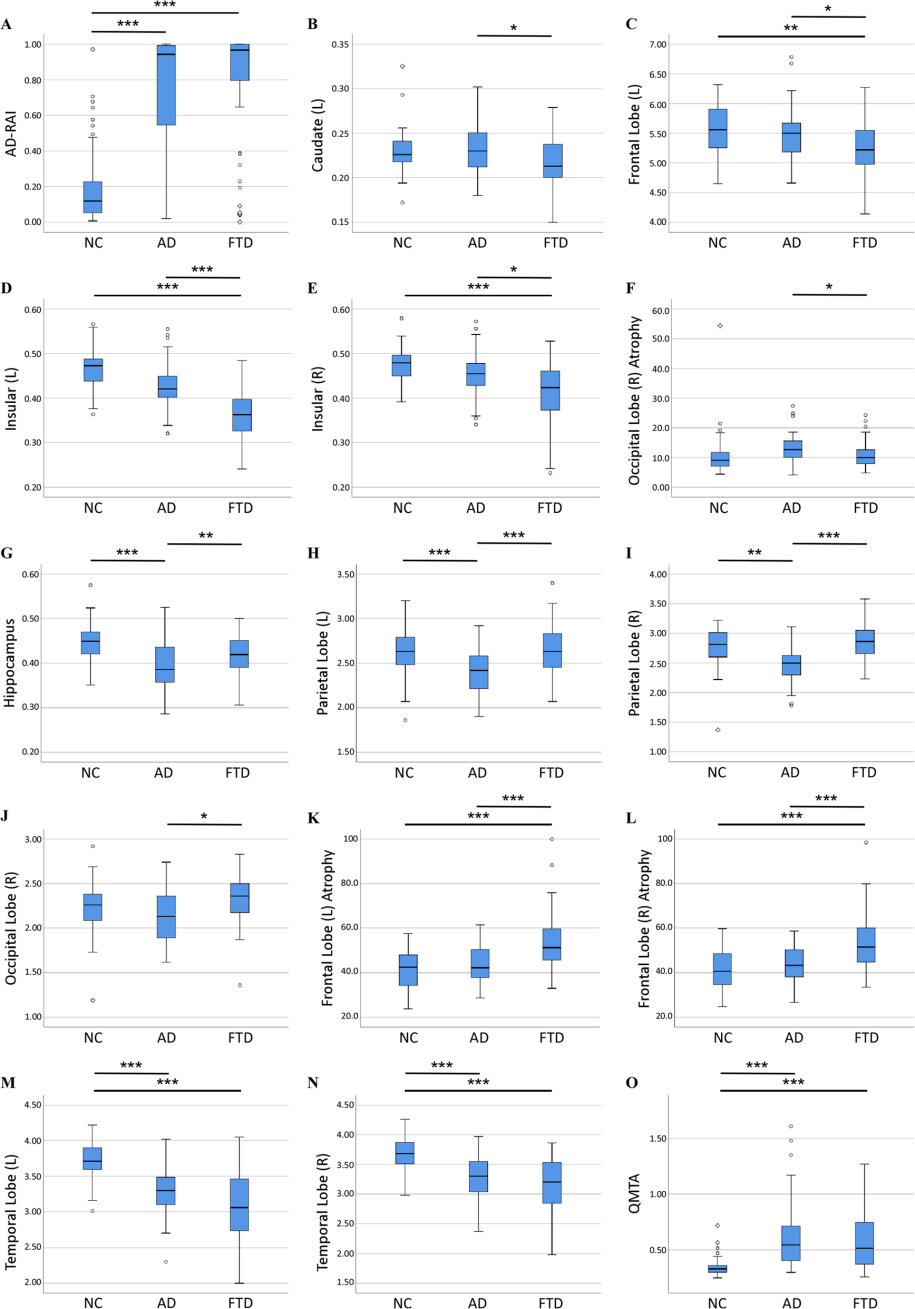
Box plots of AD-RAI and volumetric brain measures among NC, AD, and FTD groups from the NACC database. **a** AD-RAI among NC, AD, and FTD groups. **b**–**f** Volumetric brain measures which were significantly lower in the FTD group than in AD. **g**–**l** Volumetric brain measures which were significantly higher in the FTD group than in AD. **m**, **n** Relative volumes of bilateral temporal lobes and **o** QMTA among NC, AD, and FTD groups (*p < 0.05, **p < 0.01, ***p < 0.001)

**Fig. 3:**
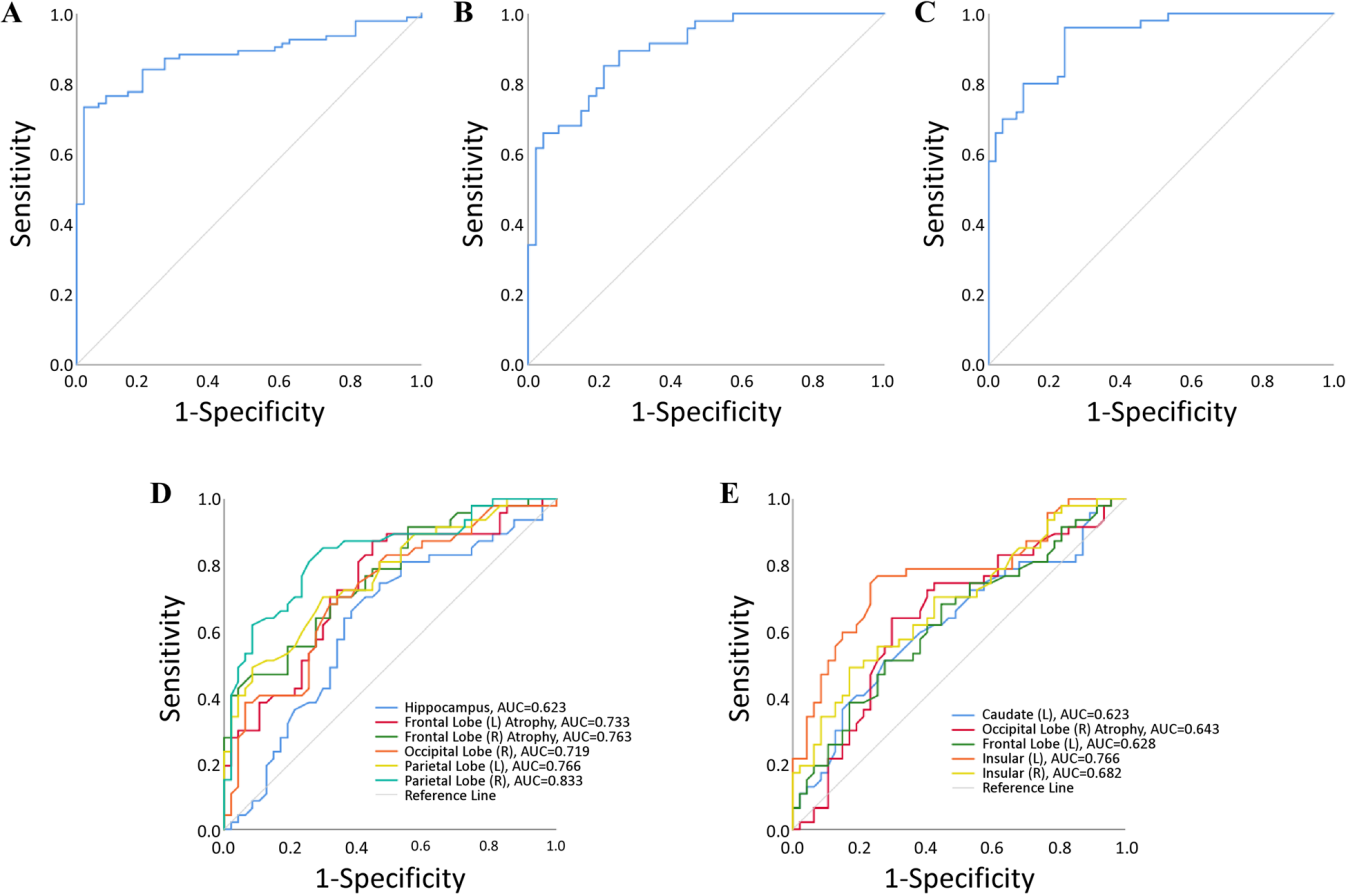
Diagnostic performance of different indexes. **a** ROC of AD-RAI using data from the NACC database, AUC = 0.88. **b** ROC of FTDI using data from the NACC database, AUC = 0.90. **c** ROC of FTDI using data from ADNI and FTDNI databases, AUC = 0.93. **d**, **e** ROC of the single volumetric brain measure which was significantly different between AD and FTD groups

Compared with the NC group, we found that the AD group showed significant (*p* < 0.05) atrophy in the brain parenchyma, bilateral hippocampi, temporal lobes, parietal lobes, amygdalae, and left cingulate lobe, and the ventricular system was significantly enlarged (see Additional Table 1). The FTD group showed significant (*p* < 0.05) atrophy in the brain parenchyma, bilateral frontal lobes, temporal lobes, insular lobes, amygdalae, and left cingulate lobe (see Additional Table 1). These findings are largely consistent with previous studies [43–45]. The AD-RAI was significantly different between NC when compared with AD and FTD, respectively (Table 2, Fig. 2a). As we expected, AD-RAI performed well (AUC = 0.88) with 97.9% sensitivity and 73.4% specificity (Fig. 3a). However, when comparing FTD with AD, AD-RAI showed no significant (*p* = 0.647) difference (Table 2, Fig. 2a).

**Table 2:** Comparison of AD-RAI and volumetric brain measures among NC, AD, and FTD groups from NACC

	NC (***n*** = 47)	AD (***n*** = 47)	FTD (***n*** = 47)	***p*** value		
				NC vs AD	NC vs FTD	AD vs FTD
AD-RAI	0.12 (0.20)	0.94 (0.51)	0.97 (0.23)	**< 0.001**	**< 0.001**	0.647
Hippocampus	0.45 (0.05)	0.39 (0.09)	0.42 (0.07)	**< 0.001**	0.446	**0.008**
Caudate (L)	0.23 (0.02)	0.23 (0.04)	0.21 (0.04)	0.491	0.836	**0.040**
Frontal lobe (L) atrophy	42.50 (14.40)	42.30 (13.30)	51.30 (14.10)	> 0.999	**< 0.001**	**0.002**
Frontal lobe (R) atrophy	40.70 (14.80)	43.40 (12.80)	51.50 (15.30)	0.647	**< 0.001**	**0.001**
Occipital lobe (R) atrophy	9.11 (4.82)	12.70 (5.90)	10.00 (5.46)	**< 0.001**	> 0.999	**< 0.001**
Frontal lobe (L)	5.56 (0.67)	5.50 (0.51)	5.22 (0.65)	> 0.999	**0.001**	**0.022**
Occipital lobe (R)	2.26 (0.31)	2.13 (0.52)	2.36 (0.34)	> 0.999	0.150	**0.022**
Temporal lobe (L)	3.71 (0.32)	3.30 (0.40)	3.06 (0.76)	**< 0.001**	**< 0.001**	0.061
Temporal lobe (R)	3.68 (0.37)	3.30 (0.55)	3.20 (0.71)	**< 0.001**	**< 0.001**	0.540
Parietal lobe (L)	2.63 (0.31)	2.42 (0.38)	2.63 (0.38)	**< 0.001**	> 0.999	**< 0.001**
Parietal lobe (R)	2.81 (0.42)	2.50 (0.37)	2.86 (0.40)	**0.005**	0.239	**< 0.001**
Insular (L)	0.47 (0.05)	0.42 (0.05)	0.36 (0.07)	0.239	**< 0.001**	**< 0.001**
Insular (R)	0.48 (0.05)	0.46 (0.05)	0.42 (0.09)	0.103	**< 0.001**	**0.012**
QMTA	0.33 (0.07)	0.55 (0.32)	0.52 (0.38)	**< 0.001**	**< 0.001**	> 0.999
MTA score	0.25 (1.00)	1.50 (1.50)	1.50 (1.50)	**< 0.001**	**< 0.001**	> 0.999

The comparison was performed with Friedman *M* test and a post hoc analysis with Wilcoxon signed-rank test with Bonferroni correction. *p* value would be highlighted in bold when it was below 0.05. The median with inter-quartile range (in bracket) of AD-RAI and the volumetric brain measures in all three groups were provided. *L* left, *R* right

### Diagnostic performance of single volumetric brain measure and FTDI

We found that the *relative volume* of the total hippocampus, left caudate, left frontal lobe, right occipital lobe, bilateral parietal lobes, bilateral insular lobes, and *atrophy* of bilateral frontal lobes and right occipital lobe were significantly (*p* < 0.05) different (Table 2, Fig. 2b–l) between AD and FTD groups. However, the temporal lobe, MTA score, and quantitative MTA (QMTA, defined as the ratio between the volumes of the inferior lateral ventricle and hippocampus using AccuBrain and Spearmen correlation of QMTA and MTA score was 0.805 [40]) of AD and FTD all showed significant difference with normal people but no significant difference existed between the two in this study (Table 2, Fig. 2m–o). As previously mentioned, among these structures, those with statistically larger volumetric brain measure in the FTD group were defined as the “FTD dominant” structures and those with statistically larger volumetric brain measure in the AD group were defined as the “AD dominant” structures, and the ratio between the two derived FTDI. When a single volumetric brain measure was used to distinguish FTD from AD in the NACC database, the AUC could only reach 0.63~0.83 (Fig. 3d, e). The parietal lobe, consistent with the previous study [43], provided the highest diagnostic accuracy. When FTDI was used for differential diagnosis of FTD and AD in the NACC database, the AUC could reach 0.90 (Fig. 3b). We chose FTDI > 1.08 as the threshold value according to the optimal Youden Index, which ensures high sensitivity (89.4%) and relatively good specificity (74.5%). When validated in the data from ADNI and FTDNI databases, FTDI performed robustly with 96.0% sensitivity and 70.0% specificity (AUC=0.93, Fig. 3c).

## Discussion

**Fig. 4:**
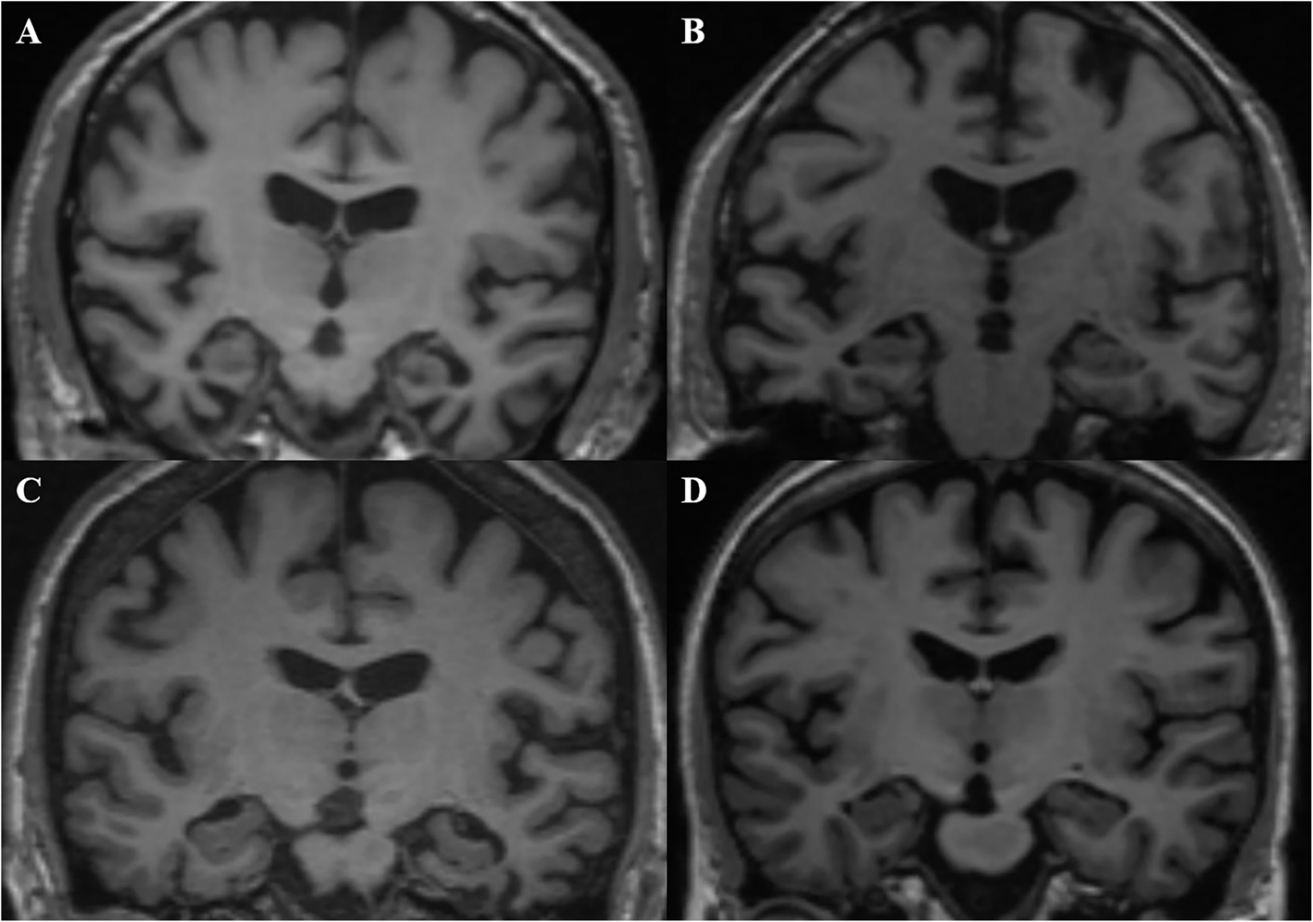
3D T1-weighted MRI scans (classical coronal section) of **a** FTD with CDR = 0.5, ID NACC204983; **b** AD with CDR = 0.5, ID NACC162576; **c** FTD with CDR = 1.0, ID NACC067187; and **d** AD with CDR = 1.0, ID NACC878860

**Fig. 5:**
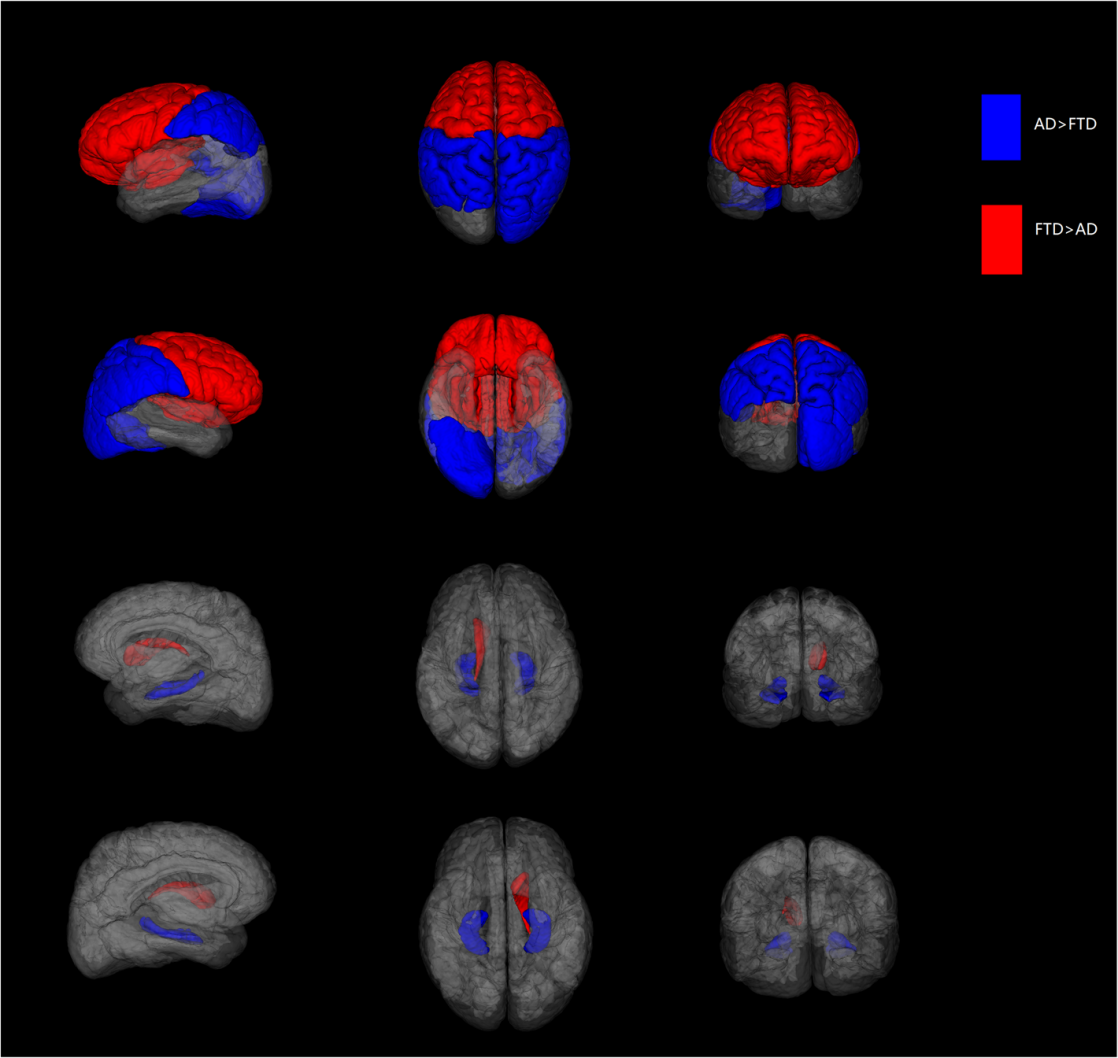
The distinct brain atrophy patterns between FTD and AD. The red color labeled the brain structures with higher atrophy in FTD including the bilateral frontal lobes, bilateral insular lobes, and left caudate. Likewise, the blue color labeled the brain structures with higher atrophy in AD including the bilateral parietal lobes, right occipital lobe, and total hippocampi

**Fig. 6:**
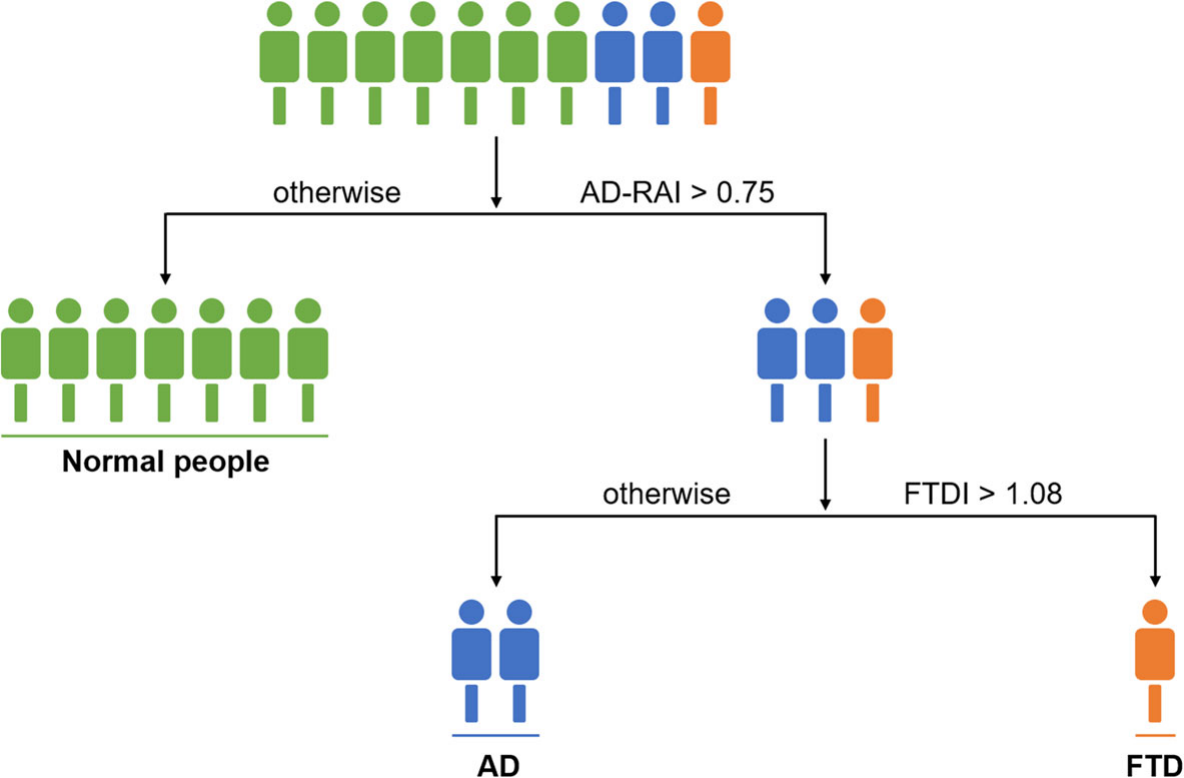
Quantitative evidence-based decision strategy to differentially diagnose AD and FTD from NC

In this study, we developed a novel MRI biomarker for differentiating AD and FTD using their dominant brain volumetric measures derived from statistical analysis of the automated and objective volumetry. The CDR scores of subjects with FTD/AD included from NACC in our study ranged mostly from 0 to 1 (> 93%) and MTA scores using visual rating scale showed no significant difference between the two groups, which means most of them experienced an early stage of AD/FTD and the atrophy patterns of the two may overlap or be too subtle (Fig. 4). Group-level comparisons were firstly applied to find the distinct brain atrophy patterns between FTD and AD (Fig. 5), and FTDI was exactly an individual index derived from those differential volumetric brain measures. By combining with the previously developed AD-RAI, an MRI-based diagnostic strategy was designed (Fig. 6) for the differentiation of FTD and AD. The FTDI model and the threshold value was determined using data from NACC and was validated in a separate cohort of data combined from ADNI and FTDNI databases. The accuracy of AD-RAI in identifying the AD and FTD from NC measured by AUC is 0.88. In this strategy, we set the threshold value of 0.75 with high sensitivity (97.9%) to reduce the rate of missed diagnosis, while maintaining a relatively high specificity (73.4%). When distinguishing FTD from AD, the AUC of FTDI was 0.90, which is comparable and even higher than previous studies [19–22, 46, 47]. The advantage of FTDI is that it is intuitively interpretable with a clear clinical meaning. Unlike complicated machine learning method or advanced imaging examinations, our work based on sMRI provided a simple logical algorithm and decision strategy to help clinicians understand and use. As the therapeutic strategy needs to be tailored for FTD and AD, the high accuracy in differentiating FTD and AD is more favored. For example, cholinesterase inhibitors effective against AD may aggravate the condition of FTD patients [4]. With this consideration, we chose a threshold value of FTDI of 1.08 with high sensitivity (89.4%) to reduce potential missed diagnosis of FTD. When validated on the independent testing data from ADNI and FTDNI databases, FTDI was proven to be satisfactorily accurate (AUC = 0.93, SEN = 96%). Our research was based on real-world data analysis and hence has high interpretability.

The ideal imaging-assisted diagnosis for clinical use should be objective, economic, simple, and efficient. Therefore, many studies adopted the automatic brain segmentation tool to identify FTD and AD through machine learning. These models need to be trained on a large number of high-quality and uniformly acquired images. Obtaining these images from a population with a low incidence of FTD will be a challenge. In addition, these methods are considered to be the “black box” and difficult for clinicians to understand, so most of them are hard to be adopted in clinical practice. In this study, we acquired enough subjects from three different well-recognized multicenter databases to ensure the robustness of the results that can be translated to the real-world application. Secondly, AccuBrain® is a National Medical Products Administration (NMPA) and CE-marked commercially automated brain segmentation tool that can be readily deployed in the clinic. Thirdly, clinicians without computer expertise can easily apply FTDI to daily clinical practice. Thus, compared with most existing MRI-based FTD classification methods, FTDI has a higher clinical application prospect.

A few studies have attempted to classify the subtypes of FTD based on MRI [20, 46, 47]. The proposed methodology in this study could be further extended into the classification of FTD subtypes. But it is beyond the scope of this study. This study, instead, is focused on a more important clinical problem, which is the differentiation of FTD as a whole with AD. However, different proportion of subtypes of the FTD spectrum disorders may result in a potential patient selection bias. FTDI is a composite index derived from several different volumetric brain measures after weighted. Preliminary validation in this work has proven its good robustness.

## Limitations

This study has several limitations. First of all, pathological diagnosis is not mandatory during subject inclusion, although it may reduce the likelihood of misdiagnosis. However, essentially, there is no recognized in vivo pathological biomarker of FTD [48, 49] and the pathological diagnosis of FTD usually comes from autopsy, which increases the difficulty of acquisition. To ensure adequate sample size, we relied more on the clinical diagnosis as indicated in the database to include the subjects. Another limitation of this study is that it was only tested and validated on three databases. Although inclusion and exclusion criteria of these databases were fairly standard and comparable to those in clinical trials, the CDR status is not reflective of the distribution of analogous patients seen in clinical practice and could bias results. However, the results of this work preliminarily showed the benefits of FTDI and our decision strategy in differentiation of FTD and AD. In addition, this study included 241 subjects in total, which is a relatively modest sample size. Before application to clinical settings, a larger dataset for further test and validation is needed. It can be difficult to collect data and maintain a relatively consistent standard, which may have a significant effect on MRI analysis. Meanwhile, due to the current limited data, the diagnostic efficacy of FTDI is unproven for patients beyond the age range specified in this study.

Although this study has these limitations, it provides a novel algorithm that takes full advantage of the different patterns of brain atrophy in AD and FTD, and it can incorporate more data to make the diagnosis more accurate. Most importantly, through this study, we came up with a method to derive individualized FTDI and provided its threshold value using the data from the NACC database and developed an MRI-based decision strategy, which is obviously more beneficial for clinicians to understand and use than complex machine learning programs.

## Conclusions

In this study, the volumetric metrics of structural MRI were investigated in a multicenter cohort of FTD, AD, and NC. These volumetric features and group-level difference were then explored to an FTD atrophy-specific index, named FTDI. Combined with the AD resemblance atrophy index, AD-RAI, we developed a strategy for identifying and differentiating FTD and AD based on MRI-induced volumetric information. Compared with visual rating and machine learning, the proposed strategy is more accurate, objective, and easier to implement.

## Supplementary Information

**Additional file 1: Additional Material 1.** Brief introduction of the databases where data were collected.

**Additional file 2: Additional Table 1.** Comparison of all the volumetric brain measures among NC, AD and FTD group from NACC. The comparison was performed with Friedman M test and a post hoc analysis with Wilcoxon signed-rank test with Bonferroni correction. *P* value would be highlighted in bold when it was below 0.05 and would not be shown if difference was not significant among the three groups. The median with inter-quartile range of AD-RAI and the volumetric brain measures in all three groups were provided. L, left; R, right.

## Data Availability

The datasets used and analyzed during the current study are available from the corresponding author on reasonable request.

## Abbreviations

AD: Alzheimer’s disease
FTD: Frontotemporal dementia
sMRI: Structural magnetic resonance imaging
bvFTD: Behavioral variant frontotemporal dementia
PPA: Primary progressive aphasia
svPPA: Semantic variant PPA
nfvPPA: Non-fluent variant PPA
NC: Normal controls
NACC: National Alzheimer’s Coordinating Center
ADNI: Alzheimer’s Disease Neuroimaging Initiative
FTLDNI: Frontotemporal Lobar Degeneration Neuroimaging Initiative
AD-RAI: AD resemblance atrophy index
FTDI: Frontotemporal dementia index
MCI: Mild cognitive impairment
CDR: Clinical dementia rating
ICV: Intracranial volume
MTA: Medial temporal atrophy
ICC: Interclass correlation coefficient
ROC curve: Receiver operating characteristic curve
AUC: Area under the curve
SEN: Sensitivity
SPE: Specificity
