# Supplementary material for: An MRI-based strategy for differentiation of frontotemporal dementia and Alzheimer’s disease
Source: Alzheimers Res Ther. 2021 Jan 12;13:23. doi: 10.1186/s13195-020-00757-5 (PMC7805212; doi:10.1186/s13195-020-00757-5)
Supplement: Supplementary file 1 — Additional file 1: Additional Material 1. Brief introduction of the databases where data were collected. [file 13195_2020_757_MOESM1_ESM.docx]

National Alzheimer’s Coordinating Center (NACC, <https://www.alz.washington.edu>) was established in 1999 and maintains a cumulative database including clinical evaluations, neuropathology data when available, and now MR imaging. the

Alzheimer’s Disease Neuroimaging initiative (ADNI, <http://adni.loni.usc.edu/>) was launched in 2003 as a public-private partnership, led by Principal Investigator Michael W. Weiner, MD. The primary goal of ADNI has been to test whether serial MRI, PET, other biological markers, and clinical and neuropsychological assessment can be combined to measure the progression of mild cognitive impairment (MCI) and early AD.

The frontotemporal lobar degeneration neuroimaging initiative (FTLDNI, <https://www.alz.washington.edu>) database was started in 2010 and the primary goals of FTLDNI were to identify neuroimaging modalities and methods of analysis for tracking frontotemporal lobar degeneration (FTLD) and to assess the value of imaging versus other biomarkers in diagnostic roles. The Principal Investigator of FTLDNI was Dr. Howard Rosen, MD at the University of California, San Francisco.
