# Supplementary material for: An MRI-based strategy for differentiation of frontotemporal dementia and Alzheimer’s disease
Source: Alzheimers Res Ther. 2021 Jan 12;13:23. doi: 10.1186/s13195-020-00757-5 (PMC7805212; doi:10.1186/s13195-020-00757-5)
Supplement: Supplementary file 2 — Additional file 2: Additional Table 1. Comparison of all the volumetric brain measures among NC, AD and FTD group from NACC. The comparison was performed with Friedman M test and a post hoc analysis with Wilcoxon signed-rank test with Bonferroni correction. P value would be highlighted in bold when it was below 0.05 and would not be shown if difference was not significant among the three groups. The median with inter-quartile range of AD-RAI and the volumetric brain measures in all three groups were provided. L, left; R, right. [file 13195_2020_757_MOESM2_ESM.docx]

**Additional Table 1. Comparison of all the volumetric brain measures among NC, AD and FTD group from NACC**

|  | **NC (n=47)** | **AD (n=47)** | **FTD (n=47)** | ***P* value** | | |
| --- | --- | --- | --- | --- | --- | --- |
|  |  |  |  | **NC vs AD** | **NC vs FTD** | **AD vs FTD** |
| Brain Parenchyma | 77.10 (4.70) | 74.10 (4.70) | 72.90 (3.10) | **< 0.001** | **< 0.001** | 0.133 |
| Hippocampus | 0.45 (0.05) | 0.39 (0.09) | 0.42 (0.07) | **< 0.001** | 0.446 | **0.008** |
| Amygdala | 0.24 (0.02) | 0.21 (0.05) | 0.23 (0.06) | **0.001** | **0.007** | > 0.999 |
| Ventricular System | 1.57 (1.20) | 2.82 (1.78) | 2.77 (1.68) | **< 0.001** | **0.003** | > 0.999 |
| Lateral Ventricle | 1.25 (1.11) | 2.50 (1.54) | 2.33 (1.49) | **< 0.001** | **0.001** | > 0.999 |
| Third Ventricle | 0.08 (0.04) | 0.11 (0.06) | 0.12 (0.06) | **< 0.001** | **< 0.001** | > 0.999 |
| Fourth Ventricle | 0.06 (0.03) | 0.06 (0.03) | 0.07 (0.02) | **-** | **-** | - |
| Inf-Lat-Vent | 0.15 (0.03) | 0.22 (0.15) | 0.23 (0.14) | **< 0.001** | **< 0.001** | > 0.999 |
| Thalamus-Proper | 0.82 (0.08) | 0.79 (0.10) | 0.79 (0.09) | **< 0.001** | 0.091 | 0.091 |
| Caudate | 0.46 (0.04) | 0.46 (0.07) | 0.43 (0.07) | **0.002** | **0.026** | > 0.999 |
| Putamen | 0.67 (0.08) | 0.65 (0.11) | 0.64 (0.08) | **0.003** | **0.004** | > 0.999 |
| Pallidum | 0.19 (0.03) | 0.19 (0.03) | 0.19 (0.03) | **0.001** | **0.012** | > 0.999 |
| Hypothalamus | 0.04 (0.01) | 0.04 (0.01) | 0.04 (0.01) | **< 0.001** | **< 0.001** | > 0.999 |
| Midbrain | 0.4 (0.04) | 0.4 (0.04) | 0.4 (0.06) | **< 0.001** | **< 0.001** | > 0.999 |
| Pons | 1.00 (0.13) | 0.99 (0.16) | 1.02 (0.13) | **< 0.001** | **< 0.001** | > 0.999 |
| Medulla | 0.30 (0.04) | 0.29 (0.03) | 0.30 (0.04) | **< 0.001** | **< 0.001** | > 0.999 |
| Cerebellum | 9.58 (0.60) | 9.07 (0.97) | 9.44 (1.08) | 0.169 | **0.030** | > 0.999 |
| Hippocampus (L) | 0.23 (0.03) | 0.19 (0.04) | 0.21 (0.04) | **-** | **-** | - |
| Hippocampus (R) | 0.22 (0.03) | 0.20 (0.04) | 0.22 (0.04) | **-** | **-** | - |
| Amygdala (L) | 0.12 (0.01) | 0.10 (0.03) | 0.11 (0.03) | **-** | **-** | - |
| Amygdala (R) | 0.13 (0.01) | 0.10 (0.03) | 0.12 (0.03) | **-** | **-** | - |
| Lateral Ventricle (L) | 0.65 (0.49) | 1.15 (0.90) | 1.08 (0.68) | **-** | **-** | - |
| Lateral Ventricle (R) | 0.58 (0.48) | 1.04 (0.81) | 1.09 (0.63) | **-** | **-** | - |
| Inf-Lat-Vent (L) | 0.08 (0.01) | 0.10 (0.07) | 0.11 (0.06) | **-** | **-** | - |
| Inf-Lat-Vent (R) | 0.07 (0.02) | 0.11 (0.07) | 0.10 (0.07) | **-** | **-** | - |
| Thalamus-Proper (L) | 0.40 (0.04) | 0.39 (0.06) | 0.39 (0.05) | **-** | **-** | - |
| Thalamus-Proper (R) | 0.41 (0.04) | 0.40 (0.05) | 0.41 (0.05) | **-** | **-** | - |
| Caudate (L) | 0.23 (0.02) | 0.23 (0.04) | 0.21 (0.04) | 0.491 | 0.836 | **0.040** |
| Caudate (R) | 0.23 (0.02) | 0.23 (0.03) | 0.22 (0.03) | **-** | **-** | - |
| Putamen (L) | 0.33 (0.04) | 0.32 (0.06) | 0.30 (0.05) | 0.053 | **0.030** | > 0.999 |
| Putamen (R) | 0.34 (0.04) | 0.33 (0.06) | 0.33 (0.05) | **-** | **-** | - |
| Pallidum (L) | 0.10 (0.01) | 0.10 (0.02) | 0.10 (0.02) | **-** | **-** | - |
| Pallidum (R) | 0.09 (0.01) | 0.09 (0.01) | 0.09 (0.02) | **-** | **-** | - |
| Frontal Lobe (L) Atrophy | 42.50 (14.40) | 42.30 (13.30) | 51.30 (14.10) | > 0.999 | **< 0.001** | **0.002** |
| Frontal Lobe (R) Atrophy | 40.70 (14.80) | 43.40 (12.80) | 51.50 (15.30) | 0.647 | **< 0.001** | **0.001** |
| Occipital Lobe (L) Atrophy | 12.90 (5.80) | 14.80 (6.90) | 14.80 (4.90) | **-** | **-** | - |
| Occipital Lobe (R) Atrophy | 9.11 (4.82) | 12.70 (5.90) | 10.00 (5.46) | **< 0.001** | > 0.999 | **< 0.001** |
| Temporal Lobe (L) Atrophy | 25.60 (8.90) | 33.40 (13.20) | 39.40 (21.10) | **< 0.001** | **< 0.001** | 0.297 |
| Temporal Lobe (R) Atrophy | 19.30 (4.90) | 26.40 (12.40) | 23.70 (13.70) | **< 0.001** | **< 0.001** | > 0.999 |
| Parietal Lobe (L) Atrophy | 41.60 (12.50) | 46.30 (14.00) | 48.30 (17.40) | 0.770 | **0.012** | 0.239 |
| Parietal Lobe (R) Atrophy | 36.60 (15.00) | 43.80 (15.60) | 43.20 (13.70) | **-** | **-** | - |
| Cingulate Lobe (L) Atrophy | 9.58 (6.76) | 11.80 (4.42) | 13.50 (9.59) | **0.006** | **0.016** | ＞0.999 |
| Cingulate Lobe (R) Atrophy | 16.80 (8.20) | 19.80 (8.90) | 19.00 (13.80) | **< 0.001** | 0.061 | 0.266 |
| Insular (L) Atrophy | 19.90 (12.60) | 29.80 (17.10) | 36.30 (18.20) | **< 0.001** | **< 0.001** | 0.080 |
| Insular (R) Atrophy | 16.50 (6.80) | 22.30 (11.50) | 21.40 (14.80) | **< 0.001** | **< 0.001** | ＞0.999 |
| Cerebellum Atrophy | 12.40 (4.50) | 10.60 (6.45) | 9.87 (3.88) | 0.061 | **< 0.001** | 0.190 |
| White Matter | 33.50 (3.10) | 33.30 (2.60) | 32.60 (2.50) | 0.836 | **0.010** | 0.190 |
| Gray Matter | 43.20 (3.60) | 40.80 (2.50) | 40.50 (1.80) | **0.001** | **< 0.001** | 0.647 |
| Frontal Lobe (L) | 5.56 (0.67) | 5.50 (0.51) | 5.22 (0.65) | ＞0.999 | **0.001** | **0.022** |
| Frontal Lobe (R) | 5.56 (0.79) | 5.42 (0.63) | 5.34 (0.68) | **0.001** | **0.007** | ＞0.999 |
| Occipital Lobe (L) | 2.53 (0.48) | 2.43 (0.37) | 2.58 (0.33) | **-** | **-** | - |
| Occipital Lobe (R) | 2.26 (0.31) | 2.13 (0.52) | 2.36 (0.34) | ＞0.999 | 0.150 | **0.022** |
| Temporal Lobe (L) | 3.71 (0.32) | 3.30 (0.40) | 3.06 (0.76) | **< 0.001** | **< 0.001** | 0.061 |
| Temporal Lobe (R) | 3.68 (0.37) | 3.30 (0.55) | 3.20 (0.71) | **< 0.001** | **< 0.001** | 0.540 |
| Parietal Lobe (L) | 2.63 (0.31) | 2.42 (0.38) | 2.63 (0.38) | **< 0.001** | ＞0.999 | **< 0.001** |
| Parietal Lobe (R) | 2.81 (0.42) | 2.50 (0.37) | 2.86 (0.40) | **0.005** | 0.239 | **< 0.001** |
| Cingulate Lobe (L) | 0.85 (0.12) | 0.78 (0.14) | 0.74 (0.08) | **0.040** | **0.001** | 0.647 |
| Cingulate Lobe (R) | 0.96 (0.15) | 0.84 (0.17) | 0.84 (0.12) | 0.117 | 0.070 | ＞0.999 |
| Insular (L) | 0.47 (0.05) | 0.42 (0.05) | 0.36 (0.07) | 0.239 | **< 0.001** | **< 0.001** |
| Insular (R) | 0.48 (0.05) | 0.46 (0.05) | 0.42 (0.09) | 0.103 | **< 0.001** | **0.012** |
| QMTA | 0.33 (0.07) | 0.55 (0.32) | 0.52 (0.38) | **< 0.001** | **< 0.001** | ＞0.999 |

The comparison was performed with Friedman M test and a post hoc analysis with Wilcoxon signed-rank test with Bonferroni correction. *P* value would be highlighted in bold when it was below 0.05 and would not be shown if difference was not significant among the three groups. The median with inter-quartile range of AD-RAI and the volumetric brain measures in all three groups were provided. L, left; R, right.
